# REsveratrol for VAscular cognitive impairment investigating cerebral Metabolism and Perfusion (REVAMP trial): a study protocol for a randomized, double-blind, placebo-controlled trial

**DOI:** 10.3389/fnut.2024.1359330

**Published:** 2024-04-23

**Authors:** Yorito Hattori, Manabu Minami, Katsuhiro Omae, Takeshi Yoshimoto, Soichiro Abe, Haruko Yamamoto, Hidehiro Iida, Masafumi Ihara

**Affiliations:** ^1^Department of Neurology, National Cerebral and Cardiovascular Center, Suita, Japan; ^2^Department of Data Science, National Cerebral and Cardiovascular Center, Suita, Japan; ^3^Turku PET Centre, University of Turku and Turku University Hospital, Turku, Finland

**Keywords:** resveratrol, carotid artery stenosis, carotid artery occlusion, vascular cognitive impairment, mild cognitive impairment, dementia, ^15^O-gas positron emission tomography

## Abstract

**Background:**

Carotid artery stenosis or occlusion (CASO) is a major cause of vascular cognitive impairment (VCI). There is currently no effective treatment for VCI induced by CASO. Resveratrol, a type of polyphenol, improves cognitive performance in rat CASO models via pleiotropic effects. Furthermore, we previously reported the longevity gene, *SIRT1*, which can be activated by resveratrol, improves cognitive and cerebral blood flow impairment in mouse CASO models by activating endothelial nitric oxide synthase. However, clinical evidence remains limited.

**Methods:**

The REsveratrol for VAscular cognitive impairment investigating cerebral Metabolism and Perfusion (REVAMP) trial is a randomized, double-blind, placebo-controlled trial involving patients with asymptomatic CASO. Each participant will receive either 150 mg/day of resveratrol or a placebo for 35 weeks. The primary objective is to determine whether resveratrol improves cognitive impairment, as assessed using the Alzheimer’s disease Assessment Scale–cognitive subscale 13. One of our secondary objectives is to determine whether resveratrol improves cerebral hemodynamic impairment as assessed via ^15^O-gas positron emission tomography. We will recruit 100 patients (50 per group).

**Discussion:**

The REVAMP trial may provide valuable insights into new therapeutic options, as multitarget neuroprotection could potentially improve cognitive function along with enhancements in cerebral hemodynamic status in patients with asymptomatic CASO.

**Clinical trial registration**: The REVAMP trial was prospectively registered in the Japan Registry of Clinical Trials (jRCTs051230013) on April 13, 2023.

## 1 Introduction

Carotid artery stenosis or occlusion (CASO), which can restrict cerebral hemodynamics, is an important cause of cerebral infarction and vascular cognitive impairment (1–4). According to the results of a meta-analysis encompassing 515 reports, it is estimated that patients with CASO aged 30–79 years make up 1.5% of the worldwide population, totaling approximately 57.79 million individuals (5). Asymptomatic moderate-to-severe carotid artery stenosis occurs in 4.2% of adults, with the prevalence increasing to 12.5% in men and 6.9% in women aged >70 years (6, 7). Furthermore, asymptomatic carotid artery stenosis impairs medication adherence, leading to failure of the primary prevention of ischemic stroke and vascular cognitive impairment (8). Consequently, the prevalence of asymptomatic CASO is higher, insidiously increasing the risk of vascular cognitive impairment, which suggests that asymptomatic CASO can no longer be considered truly asymptomatic but rather “symptomatic.” The undetected cognitive morbidity within such a substantial population carries potentially significant public health implications (2). However, preventive or therapeutic medication targeting vascular cognitive impairment with CASO has not been established.

Resveratrol (3, 5, 40-trihydroxy-trans-stilbene) is a type of polyphenol, which is naturally found in the skin of red grapes, red wine, blueberries, peanuts, and Japanese knotweed (9). It plays a key role in preventing various human diseases (including brain diseases) through its pleiotropic effects (10). Basic studies using rodent models have revealed that resveratrol improves cognitive performance in rat models of bilateral common carotid artery occlusion, which mimics chronic cerebral hypoperfusion. This improvement is achieved by decreasing oxidative stress, reducing inflammation, and promoting autophagy (11–13). Furthermore, resveratrol has been proven to cross the blood–brain barrier (14). We reported that the nicotinamide adenine dinucleotide (NAD^+^)-dependent longevity gene *SIRT1*, which can be activated by resveratrol (15), revealed improvements in cognitive performance and cerebral blood flow (CBF) via deacetylation of endothelial nitric oxide synthesis (eNOS) deacetylation in a mouse CASO model (16, 17). However, clinical evidence remains limited as there are only a few reports demonstrating positive effects on cognitive function in healthy subjects. In one study, the daily intake of oral resveratrol at a daily dose of 200 mg/day for 26 weeks significantly improved memory and hippocampal functional connectivity in healthy participants aged 50–75 years (9). Another study involving healthy postmenopausal women aged 45–85 years who received resveratrol at a daily dose of 150 mg/day for 14 weeks reported significant improvements in verbal memory and overall cognitive function (18). Long-term supplementation with resveratrol may be tolerable. Previous reports of resveratrol administration for 12 months have revelaed several adverse events, including exacerbation of gastric reflux, itching, menstrual changes, prolapsed bladder, and scheduled heart valve stent insertion and left eye operation. However, it is important to note that these events were not necessarily directly attributable to resveratrol supplementation at a dose of 150 mg/day (19, 20). Thus, the efficacy of resveratrol in patients affected by asymptomatic CASO remains unclear.

Before the start of the REsveratrol for VAscular cognitive impairment investigating cerebral Metabolism and Perfusion (REVAMP) trial, our preliminary retrospective observational study was conducted between July 2020 and March 2022 at the National Cerebral and Cardiovascular Center (NCVC) in Japan. The study involved 38 patients with asymptomatic CASO in the resveratrol-treated group and 44 patients with asymptomatic CASO in the nonresveratrol-treated group who underwent neuropsychological tests, such as the Alzheimer’s Disease Assessment Scale–cognitive subscale 13 (ADAS-Cog) (approval number of NCVC Research Ethics Committee: R20113). The mean observational period for the resveratrol-treated and nonresveratrol-treated groups was 223 ± 65 and 246 ± 87 days, respectively. We observed a significantly improved total ADAS-Cog score in the resveratrol group (−0.77 ± 1.88, 0.55 ± 1.74; *p* = 0.006) and an intergroup difference in the total ADAS-Cog scores of −2.35, calculated from the pooled variance estimates standard deviation of 3.86. Among the 82 patients, 79 underwent neuropsychological tests, such as ADAS-Cog and Montreal Cognitive Assessment (MoCA) and ^15^O-gas positron emission tomography (PET); 36 received resveratrol and 43 did not. Long-term resveratrol treatment significantly improved the memory domain and total score in the ADAS-Cog and the visuospatial/executive function in the MoCA. Furthermore, CBF improved in the anterior circulation territory and thalamus. No adverse events were observed (21).

Therefore, we hypothesized that long-term intake of resveratrol yielded beneficial effects on cognitive improvement accompanied by enhanced cerebral blood flow via pleiotropic effects such as activation of NAD^+^/SIRT1/eNOS axis, and decrease of oxidative stress and inflammation in patients with asymptomatic CASO. We aimed to prospectively determine whether the long-term clinical application of resveratrol at a daily dose of 150 mg improves cognitive impairment and the cerebral hemodynamic state, as assessed by ^15^O-gas PET, in patients with asymptomatic CASO. Herein, we provide a comprehensive description of the protocol for a clinical trial aimed at assessing the safety and efficacy of resveratrol administered at a daily dosage of 150 mg for patients with asymptomatic CASO.

## 2 Methods and analysis

### 2.1 Trial design

**Figure 1:**
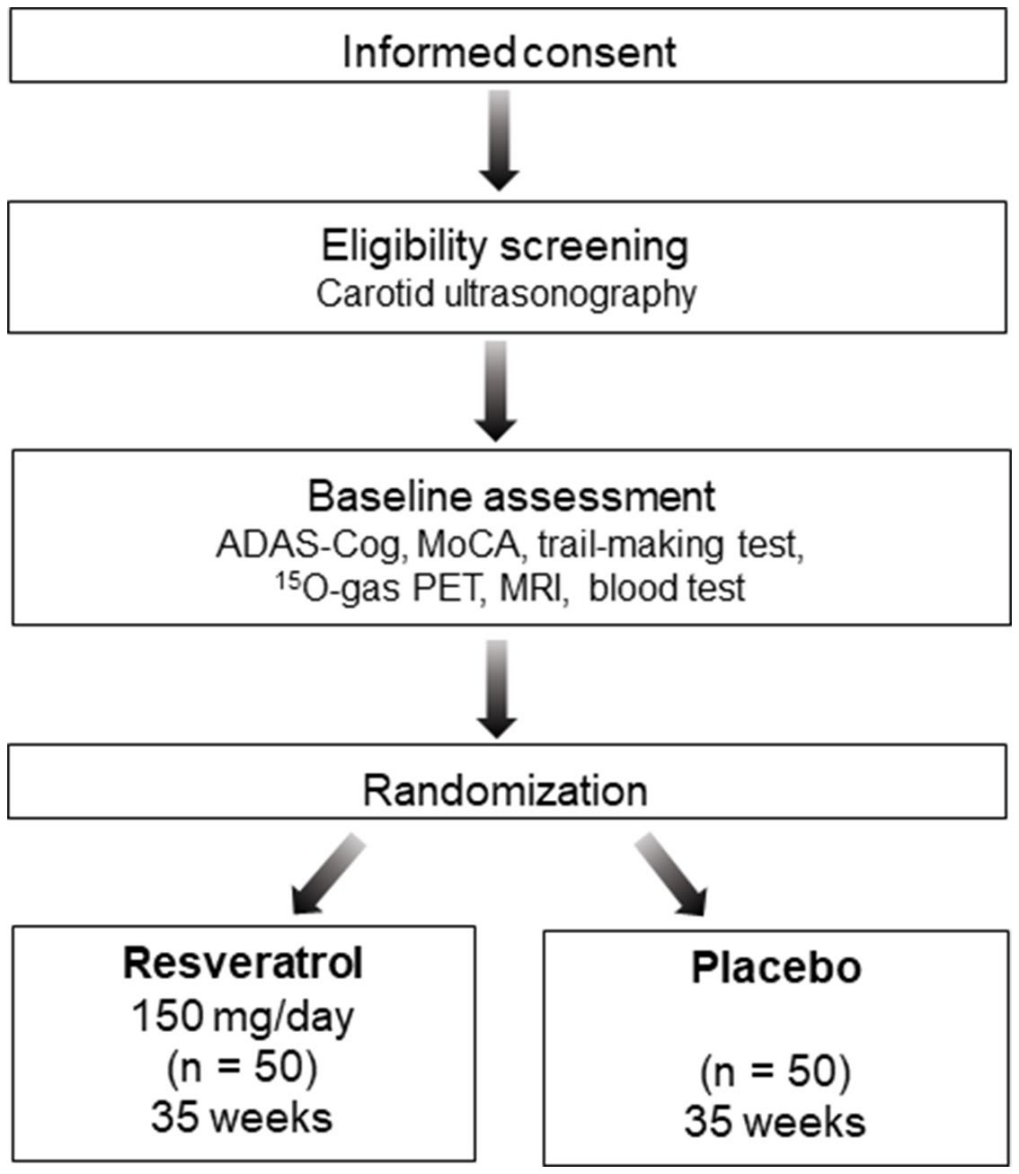
Schematic flow diagram of the REVAMP trial’s participant enrollment process. ADAS-Cog, Alzheimer’s disease Assessment Scale–cognitive subscale 13; MoCA, Montreal Cognitive Assessment; PET, positron emission tomography; MRI, magnetic resonance imaging.

**Figure 2:**
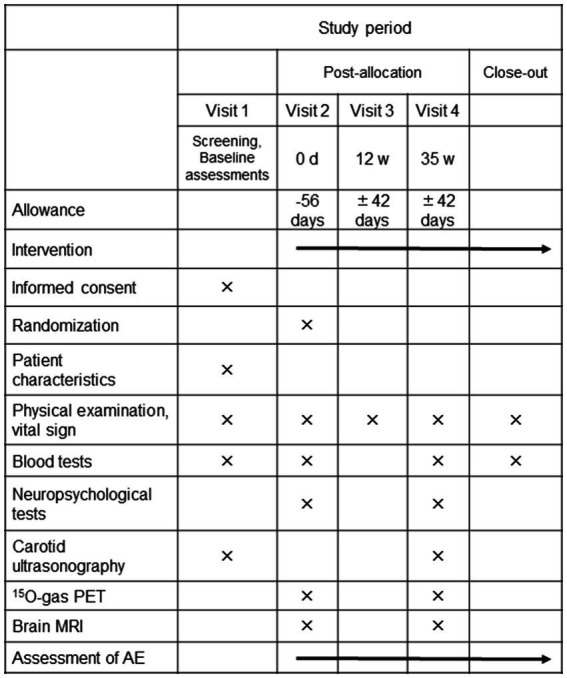
The schedule of interventions and assessments of the REVAMP trial. PET, positron emission tomography; MRI, magnetic resonance imaging; AE, adverse events.

**Figure 3:**
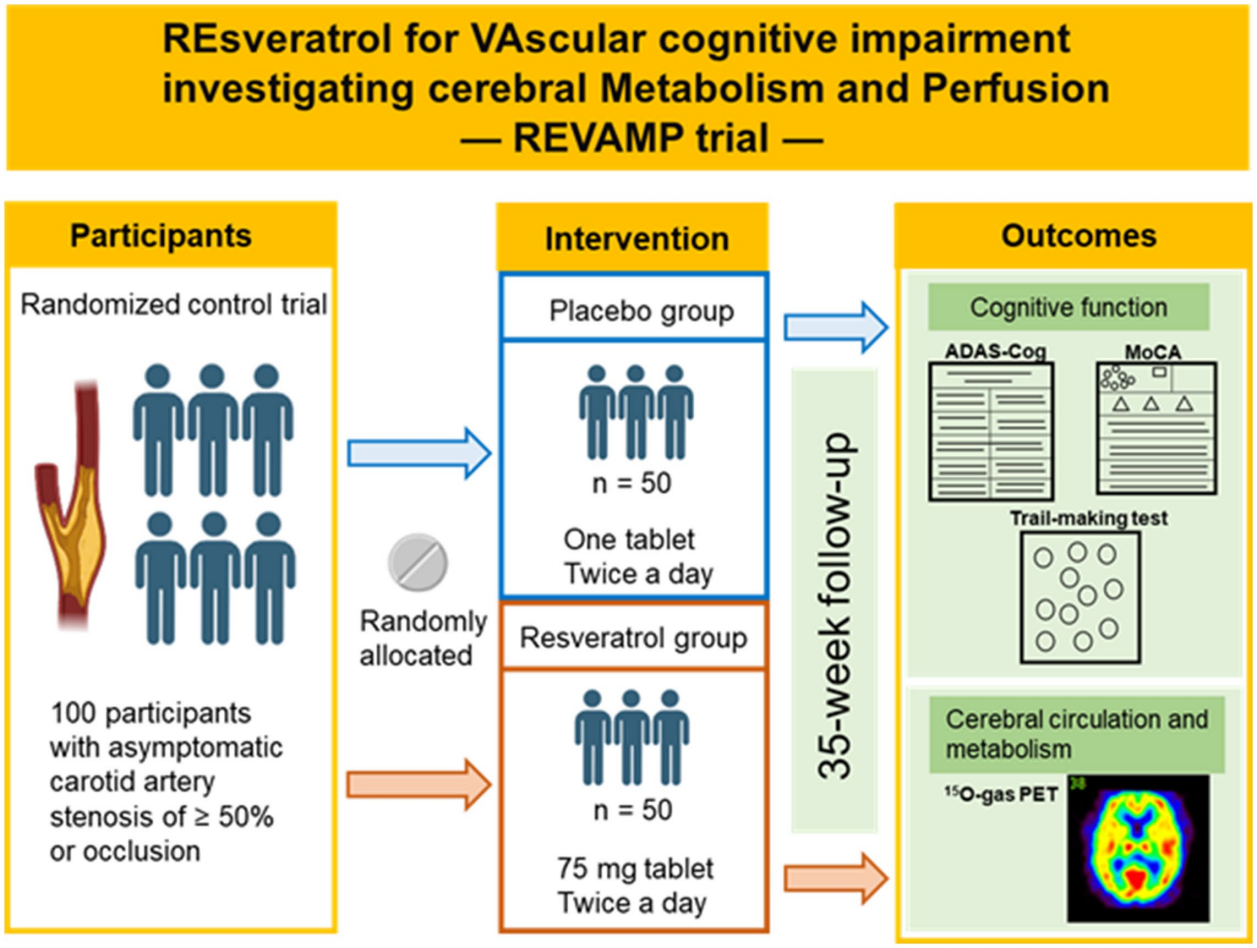
Outline of the REVAMP trial. The schematic illustration was created with Biorender.com.

The REVAMP trial is an investigator-initiated, randomized, double-blind, placebo-controlled study that aims to investigate the safety and efficacy of the long-term intake of resveratrol 150 mg/day in patients with asymptomatic CASO and will be performed at the NCVC. One hundred participants will be randomly assigned to either the resveratrol or placebo control group in a 1:1 ratio (Figures 1, 2). During the REVAMP trial, participants will be given either resveratrol or a placebo for 35 weeks (Figure 3).

The physicians involved in the study will obtain written informed consent for the trial and the NCVC biobank from all trial patients before participating in the study. In case of any revision in the written informed consent form, the physicians involved in the study will explain the revised procedure again to the participants, revise the written informed consent form, and obtain the subject’s voluntary consent for continuation of participation.

### 2.2 Study objectives

The primary objective will be change of ADAS-Cog total score from baseline to assess the effectiveness of resveratrol in preserving or improving cognitive function in patients with asymptomatic CASO. The principal secondary objectives will be to evaluate the impact of resveratrol on the following: (i) the preservation or enhancement of cognitive function, as evaluated by a change in the total MoCA score and the trail-making test score from baseline; (ii) the preservation or improvement of cerebral hemodynamic parameters, including CBF, oxygen extraction fraction (OEF), and cerebral metabolic rate of oxygen (CMRO_2_) as measured by ^15^O-gas PET (Table 1).

**Table 1:** Primary and secondary objectives.

Primary objective
Change of ADAS-Cog total score from baseline to 35 weeks.
Secondary objectives
1.Changes of MoCA total score and trail making test from baseline to 35 weeks. 2.Change of CBF examined by brain ^15^O gas-PET from baseline to 35 weeks. 3.Change of OEF examined by brain ^15^O gas-PET from baseline to 35 weeks. 4.Change of CMRO_2_ examined by brain ^15^O gas-PET from baseline to 35 weeks. 5.Volume or grade changes of white matter hyperintensities examined by brain magnetic resonance imaging (MRI) from baseline to 35 weeks. 6.Number changes of cerebral microbleeds examined by brain MRI from baseline to 35 weeks. 7.Change of systolic and diastolic blood pressure from baseline to 35 weeks.

### 2.3 Inclusion criteria

The REVAMP trial will be conducted by investigators at the NCVC. We will include the following categories of participants: (i) patients aged ≥20 years and < 90 years at the time of consent; and (ii) individuals with asymptomatic carotid artery stenosis of ≥50%, or occlusion. A peak systolic velocity of ≥130 cm/s at a stenotic lesion, as measured by carotid Doppler ultrasonography, indicated a stenotic diameter of approximately ≥50% (22). The asymptomatic status will be confirmed based on the absence of stroke or transient ischemic attack caused by the development of a carotid lesion within 6 months (3). Patients with hypertension, cardiovascular diseases, or diabetes mellitus will not be excluded. Details of our eligibility criteria are presented in Table 2.

**Table 2:** Eligibility criteria.

Inclusion criteria
1.Patients aged ≥20 years and < 90 years. 2.Patients with asymptomatic carotid artery stenosis of ≥50% or occlusion. * Definition of ≥50% stenosis: 130 cm/s or faster of peak systolic velocity at a stenotic lesion examined by carotid ultrasonography. 3.Patients who gave their written informed consent.
Exclusion criteria
1.Severely demented patients who fail to undergo neuropsychological examinations. 2.Patients taking donepezil, galantamine, rivastigmine, or memantine. 3.Patients already diagnosed with secondary dementia (Parkinson’s disease, Huntington’s disease, normal-pressure hydrocephalus, progressive supranuclear palsy, multiple system atrophy, multiple sclerosis, head injury with sequelae, neurosyphilis, hypothyroidism, vitamin B1/B12 deficiency, and folic acid deficiency). 4.Patients with ≥50% stenosis or occlusion of the intracranial basilar artery and vertebral artery on brain magnetic resonance angiography. 5.Patients with a past history of intrinsic psychiatric disease or alcohol or drug dependence within 48 weeks before giving their informed consent. 6.Patients with severe pulmonary diseases (e.g., under home oxygen therapy). 7.Patients with a history of dose change in statins, PCSK9 inhibitors, intestinal cholesterol transporter inhibitors, and phosphodiesterase inhibitors within 4 weeks before giving their informed consent. 8.Patients who did not cease the intake of resveratrol or nicotinamide mononucleotide at least 4 weeks prior to the administration of the experimental drugs if the patients already consume resveratrol or nicotinamide mononucleotide. 9.Patients with severe renal dysfunction (eGFR <15 mL/min/1.73m^2^ or hemodialysis). 10.Patients who experienced malignancy within the past 5 years. 11.Pregnant and breastfeeding women or patients who do not agree to use appropriate contraception. 12.Patients participating in or planning to participate in other clinical trials using other medicines or medical devices.

### 2.4 Screening procedures

Before enrollment, all potential participants will undergo an eligibility screening process by reviewing their medical charts to check the results of carotid ultrasonography. Personal information of potential and enrolled patients will be shared in a database accessible only within the project group to personnel responsible for patient inclusion, to protect confidentiality before, during and after the trial. At the first visit, the physicians involved in the study will provide detailed information about the REVAMP trial to the patients and/or their families and obtain written informed consent. Participants will be informed in the consent forms that they have the right to withdraw from the trial without any negative consequences. The attending physician will conduct a physical examination of all participants, and the clinical research coordinators (CRCs) will collect necessary clinical data such as height, body weight, vital signs, medical history, supplements, and demographics.

### 2.5 Baseline assessments

After the initial screening, all participants will undergo neuropsychological assessments such as ADAS-Cog, MoCA, and the trail-making test. They will also undergo ^15^O-gas PET and brain magnetic resonance imaging, randomized at visit 2 (Figure 2). Blood samples for analyses will be collected before taking either resveratrol or placebo tablets and stored in the NCVC biobank. The tablets will be then distributed to participants after randomization (See the Randomization, allocation, and blinding section) by each physician involved in the study. To ensure proper medication adherence, all participants will be instructed to fill out a schedule book specific to the REVAMP trial every day and return the remaining tablets at each visit.

### 2.6 Follow-up assessments

All participants will be required to visit the NCVC at weeks 12 and 35, which will be the final visit. During the week 35 visit, carotid ultrasonography, neuropsychological assessments, ^15^O-gas PET, and brain magnetic resonance imaging will be conducted, and blood samples will be collected and stored in the NCVC biobank. At weeks 12 and 35 (Figure 2), each physician involved in the study will conduct a physical examination, and the CRCs will conduct interviews to inquire about changes in prescription, over-the-counter medications and supplements, and occurrence of adverse events. To promote participant retention and complete follow-up, the CRCs will call the participants at least once a month to check whether the participants are continuing taking the resveratrol or placebo tablets.

### 2.7 Randomization, allocation, and blinding

The physicians involved in the study will generate the allocation sequence, enroll patients, and assign patients to interventions. Registered patients will be assigned randomly to either the resveratrol group or the control group. The assignment method will be minimization, and the stratification factors considered will be sex, the presence of left CASO, and a total score of ADAS-Cog ≥15. The randomization process will be executed using computer-generated random allocation treatment codes. Both patients and physicians will remain blinded to the assigned therapy throughout the study. The randomization list will be maintained by an independent investigator who is not involved in patient care, assessment, data collection, or analysis. Emergency unblinding will only occur if the principal investigator deems it necessary to reveal the assigned intervention to manage any potential adverse events among participants.

### 2.8 Interventions

Nature Holdings Co., Ltd. in Japan will supply standardized resveratrol and placebo tablets. The resveratrol tablets will be manufactured using 99% pure synthetic trans-resveratrol (resVida™, DSM Nutritional Products Ltd., Kaiseraugst, Switzerland), comply with Good Manufacturing Practice. The visual appearance of the placebo tablets was identical to that of the resveratrol tablets. Participants in the resveratrol group will be administered one 75-mg tablet of resveratrol twice daily (in the morning and evening) for 35 weeks. Conversely, those in the control group will receive one placebo tablet twice daily (also in the morning and evening) during the same period. Participants will consume one tablet with water. The physicians involved in the study or CRCs will maintain a comprehensive tablet inventory record, which will include information on the supply, receipt, disposal, and return of the trial tablets.

Discontinuance criteria will be as follows: (i) severe renal dysfunction (estimated glomerular filtration rate of <15 mL/min/1.73m^2^); (ii) severe gastrointestinal symptoms requiring emergent treatments; (iii) initiation of taking resveratrol or nicotinamide mononucleotide; (iv) initiation of taking antidementia drugs; and (v) administration of carotid endoarterectomy (CEA) or carotid artery stenting (CAS). Relevant concomitant care and interventions that are prohibited during the trial will be (i) resveratrol or nicotinamide mononucleotide; (ii) antidementia drugs; and (iii) CEA or CAS.

A restricted medication will be allowed if the doses are unchanged during the trial. Statins may improve anti-inflammatory, antithrombotic, and endothelial functions (23), and phosphodiesterase inhibitors may contribute to improvements in cerebrovascular regulation (24), potentially increasing CBF. Therefore, statins and phosphodiesterase inhibitors are included in the restricted medication list. Other medications or interventions may be used without restriction. Nonsteroidal anti-inflammatory drugs (NSAIDs) will not be included in the restricted medication list. NSAIDs used for <24 months do not affect cognitive function (25).

### 2.9 Neuropsychological examination

Only psychologists who have substantial experience and are blinded to the intervention groups will be authorized to perform cognitive assessments, including ADAS-Cog, MoCA, and the trail-making test.

### 2.10 ^15^O-gas PET measurements

All patients will undergo a series of ^15^O-gas PET examinations to assess CBF, CMRO_2_, and OEF. Radioactive ^15^O will be produced by accelerating a deuteron (d) beam via the 14 N (d,n) ^15^O nuclear reaction using a cyclotron (CYPRIS HM-12, Sumitomo Heavy Industry, Tokyo, Japan). Further, 0.3% oxygen (O_2_) in the nitrogen (N_2_) target will be used to produce the ^15^O–O_2_ and ^15^O–carbon monoxide (CO) gasses and 1.0% carbon dioxide (CO_2_) in the N_2_ target gas to produce the ^15^O–CO_2_ gas.

A PET scanner (Biograph mCT, Siemens Healthinier, Erlangen, Germany) will be used in the study. A PET scan was initiated 3 min after the 2-min inhalation of ^15^O–CO for 4 min. An additional dynamic PET scan was performed for 8 min during the sequential inhalation of ^15^O-O_2_ and ^15^O–CO_2_ gasses for 1 min each at a 4.5-min interval. The radiochemical purity was confirmed to be >99% before each radio gas inhalation in every patient via two-channel rapid gas chromatography (Micro 990, Agilent Technologies, Inc., Santa Clara, USA), as described in a previous study (26).

PET images will be reconstructed using vendor software, with an adequately selected methodology that considers the presence of gaseous ^15^O–radioactivity surrounding the face during images will require following the 2-min inhalation of the ^15^O–carbon monoxide gas (27). CBF and OEF images will be calculated using the previously validated dual-table autoradiography technique (28). Then, the functional images of CMRO_2_ will be calculated as follows:


$${\mathrm{CMRO}}_{2}=\left[O_{2}\right]a\times 1.39\times \%Sat\times Hb$$

[O_2_]a, the oxygen content in the arterial blood; %Sat, %saturation of oxygen in the arterial blood; Hb, the hemoglobin concentration in units of (mL/g); 1.39, the maximum amount of oxygen bound to the unit Hb mass (mL/min).

according to a previously validated technique (i.e., dual-table autoradiography technique) (28). The arterial input function will be obtained from the radioactivity concentration in the arterial blood that was continuously withdrawn from the brachial artery (28). The metabolized ^15^O-water in the arterial blood generated from ^15^O–O_2_ will be estimated by modeling physiological oxygen metabolism (29).

### 2.11 Sample size estimates

Referring to aforementioned our preliminary study performed at the NCVC between July 2020 and March 2022, we observed an intergroup difference in the total ADAS-Cog scores of −2.35 with a standard deviation calculated from the pooled variance estimates of 3.86. In line with previous studies (30–32), the effect size, calculated as | − 2.35/3.86|, was deemed sufficient to detect a clinically meaningful difference. Consequently, the sample size calculation for testing the hypothesis of the superiority of resveratrol treatment over nontreatment, with a certain degree of power, was conducted.

Since the total ADAS-Cog score at baseline is expected to correlate with the change in total score, between-group comparisons will be made via the analysis of covariance using the total ADAS-Cog score at baseline as the covariate when analyzing the primary endpoint. Under this assumption, the minimum number is 45 subjects per group based on the number of subjects required for a two-sided significance level of 5% and 80% power. However, since point estimates of correlation coefficients assessed by the limited available data are difficult to refer to, we conservatively assumed that there was no correlation.

Considering the basis for the above calculation and the difference between the dose in the resveratrol group in the REVAMP trial and the dose in the resveratrol group in the preliminary study, the actual power in the REVAMP trial is expected to be sufficiently higher than assumed. Therefore, we will recruit 100 patients (50 patients per group), considering a dropout rate of 10%. A press release notifying about start of the REVAMP trial at NCVC was issued on the website of NCVC to reach the target sample size.

### 2.12 Data collection

Web-based electronic data capture system with secure and restricted access will be used to collect clinical data obtained from patient medical records. Geographic data, medical history, medication information, laboratory data, and findings of neuropsychological tests and carotid ultrasonography will be stored in the system. Data will only be de-identified for analysis after this study. Shido Co., Ltd., a contract research organization, assigned to data management will perform quality control at each step of data handling to ensure the reliability of all data related to the trial, and maintain the web-based electronic data capture system.

### 2.13 Data monitoring

The individuals assigned to data monitoring will be responsible for safeguarding the interests of trial participants, evaluating the safety and efficacy of the interventions throughout the trial, and monitoring the overall conduct of the trial. A formal data monitoring committee will not be set up because (a) it is known that long-term resveratrol administration has minimal risks (19, 20); (b) asymptomatic CASO is not a life-threatening disease; (c) interim analysis will not be planned; and (d) vulnerable participants such as pediatric patients, patients with severe mental retardation and severely demented patients who fail to undergo neuropsychological examinations will not be included.

### 2.14 Statistical analysis

We will analyze a full analysis set (FAS), which is defined as data obtained from registered patients who will receive at least some of the assigned treatment and complete at least one assessment at the end of the 35 weeks. However, patients found to be ineligible after the registration will be excluded from the full analysis set. For the primary objective, the changes in total ADAS-Cog scores from the baseline to 35 weeks after starting protocol treatment will be tested using the analysis of covariance with covariates including the baseline ADAS-Cog total score, the existence of left CASO, and sex to assess the improvement in cognitive function brought about by the treatments In addition, the same analysis will be also performed on the per protocol set (PPS) as a supplementary analysis to confirm the robustness of the main analysis using the FAS. The PPS will not include the following categories of participants: (a) patients who will receive the prohibited concomitant care and interventions as outlined in the section 2.8 Intervention; (b) patients who will not attend visits at 35 weeks within the specified allowance; and (c) less than 75% of medication adherence. The REVAMP trial will judge the efficacy of resveratrol using these analyses. Changes in CBF, CMRO_2_, and OEF assessed by ^15^O–gas PET, white matter volume on brain magnetic resonance imaging and blood pressure from the baseline to 35 weeks will be also analyzed with the same procedure as the primary objective.

Changes from the baseline within a group will be analyzed using paired *t*-test or Wilcoxon signed-rank test, taking into account the data distribution, which Shapiro–Wilk test will be used to assess. Regarding other evaluation items, for continuous variables, the changes from baseline at each follow-up point will be evaluated using either paired *t*-test or Wilcoxon signed-rank test based on the data distribution. Comparisons do not take multiplicity into account. Student’s *t*-test or Wilcoxon rank-sum test will be used to evaluate the difference between the two groups based on the data distribution. For categorical variables, Fisher’s exact or χ^2^ test will be used. All reported *p*-values will be two-tailed, and *p* < 0.05 will be considered statistically significant.

### 2.15 Exploratory analysis

An exploratory analysis will be conducted to assess the following issues: (a) temporal changes in plasma NAD^+^ or the NAD/NADH ratio; (b) temporal changes in blood SIRT1 protein concentration or activity; (c) temporal changes in blood eNOS protein concentration; (d) associations between plasma resveratrol and its metabolite concentrations and cognitive function.

### 2.16 Safety profile

If an adverse event occurs, the physicians involved in the study will treat the patient appropriately in terms of safety and report the details of the event. If a severe adverse event occurs, the principal investigator must report it to the Director General of the NCVC and the NCVC Clinical Research Review Board. If the adverse event is caused by protocol treatment, follow-up and best medical treatment will be performed. The patient will be compensated for any study-related injuries by insurance.

## 3 Discussion

Resveratrol holds promise as a novel treatment for cognitive impairment resulting from asymptomatic CASO, with its potential mechanisms primarily centered around the restoration of cerebral hemodynamic conditions, in which the NAD^+^/*SIRT1*/eNOS axis is presumed to play a pivotal role. However, it is noteworthy that CEA and CAS do not exhibit clear effects on cognition, despite the hypothesis that improved cognitive function may be associated with enhanced cerebral perfusion (33–36). To date, three mechanisms have been suggested to explain cognitive impairment during carotid revascularization. First, operative ischemia including subclinical cerebral microembolic patterns occurring during revascularization, especially in CAS, may worsen neuropsychological function (37). Second, intraoperative and postoperative hypoperfusion due to vessel clamping or ballooning might induce cognitive impairment (38). Third, cognitive deterioration might also contrarily occur in the context of cerebral hyperperfusion (38). A meta-analysis (39) has also indicated that CAS and CEA do not provide significant improvement in patients with cognitive dysfunction. To prevent cognitive decline in patients with CASO, it is imperative to ensure cerebrovascular integrity while minimizing the risk of perioperative silent microembolic cerebral injury and maintaining perioperative appropriate cerebral perfusion levels. Additionally, there is currently a paucity of evidence supporting the effectiveness of antithrombotic drugs in preventing vascular cognitive impairment (40). As of now, there is no promising preventive or treatment strategy available for addressing CASO-related vascular cognitive impairment.

The successful improvement of cerebrovascular deterioration in rodent models through resveratrol treatment was first published in 2014. Resveratrol demonstrated its ability to rescue neurovascular coupling by reducing oxidative stress and enhancing vascular endothelial function in 24-month-old mice (41). Subsequently, several other *in vivo* studies performed on rodent CASO models have demonstrated that resveratrol exerts pleiotropic effects. These effects include the activation of autophagy and the suppression of oxidative stress by inhibiting the expression of AKT/mechanistic target of rapamycin signaling pathway-related proteins, resulting in the reduction of oxidative stress-induced neuronal damage and neuronal apoptosis (11). Additionally, resveratrol improves cognitive function through the activation of the protein kinase A and cyclic AMP-responsive element-binding protein pathway (12), and reduces inflammatory responses by downregulating the stimulator of interferon genes/TANK-binding kinase 1/interferon regulatory factor 3 signaling (13). Thus, resveratrol demonstrates these pleiotropic effects, in addition to NAD^+^/*SIRT1*/eNOS activation, in rodent CASO models. Clinically, oral resveratrol supplementation at a dose of 30 mg/day can improve endothelium-dependent vasodilation, as demonstrated by significant increases (approximately 1.5 times) in flow-mediated dilation relative to placebo (42). Therefore, resveratrol at a dose of at least 30 mg/day can activate systemic eNOS. Furthermore, resveratrol oral supplementation was associated with significant improvements in cognitive function and CBF (21). Therefore, resveratrol oral supplementation at a dose of 30 mg/day would be sufficient to modulate cerebrovascular homeostasis.

In the past, resveratrol may have promoted atherosclerosis in rabbits with hypercholesterolemia (43). However, resveratrol currently has gained increased attention because of its beneficial effects on improving metabolism in endothelial and vascular smooth muscle cells in many preclinical and clinical studies on atherosclerosis, metabolic disease, hypertension, and ischemia (44).

Resveratrol has a short initial half-life (approximately 8–14 min) and is extensively metabolized in the body (45). In contrast, the serum half-life of resveratrol metabolites was approximately 9.2 h, indicating that exposure to the metabolites is much higher than that for unchanged resveratrol. Baur and Sinclair suggest that the low bioavailability of resveratrol has led to speculation that its metabolites may retain some activity (45). In support of this, first, resveratrol metabolites are capable of activating SIRT1 (46), which is widely expressed in endothelial cells (47), leading to the promotion of endothelium-dependent vascular relaxation by activating eNOS (48). NO produced by eNOS plays a crucial role in maintaining vascular/endothelial integrity. Specifically, NO inhibits RhoA activity and protects endothelial barrier function by S-nitrosylation of RhoA (Cys^16^, Cys^20^, and Cys^159^) (49). Second, resveratrol has been proven to cross BBB (14), which may lead to ameliorate neuroinflammation after crossing BBB. Resveratrol metabolites demonstrate anti-inflammatory potential by counteracting an inflammatory challenge in an *in vitro* study using U-937 macrophages as an immune-competent model system (50). Furthermore, X-ray structural and computational modeling results suggest that resveratrol metabolites also exhibit anti-inflammatory effects by inhibiting the activity of cyclooxygenase through binding to the active arachidonic acid sites of cyclooxygenase (46). Consequently, resveratrol metabolites may contribute to the inhibition of neuroinflammation. However, the detailed *in vivo* mechanisms are still limited. These findings may explain why several clinical studies using resveratrol have revealed improvements in cognitive function and cerebral circulation (9, 18, 21).

In this trial, we will be using resveratrol at a dosage of 150 mg/day. Previous randomized controlled trials involving the administration of 200 mg/day for 26 weeks (43) and 150 mg/day for 14 weeks (18) to healthy individuals showed cognitive improvement without any adverse events. In contrast, when 500–2000 mg/day of resveratrol was administered for 52 weeks to individuals with mild-to-moderate Alzheimer’s disease, it did not result in significant cognitive improvement (51). In an open-label trial, 5 out of 24 patients with multiple myeloma (20.8%) who received 5,000 mg/day of resveratrol for 20 days experienced severe renal failure (52). Therefore, this drug has been evaluated in Europe by the European Food Safety Authority, which concluded that the 150 mg/day dosage is safe for adults to consume (53). Therefore, the recommended daily intake of 150 mg is considered to be appropriate.

In conclusion, the results of the REVAMP trial may offer insights into novel therapeutic avenues as multitarget neuroprotection may lead to improvements in cognitive function alongside enhancements in cerebral hemodynamic conditions for patients with asymptomatic CASO.

## Ethics statement

The studies involving humans were approved by National Cerebral and Cardiovascular Center Clinical Research Review Board. The studies were conducted in accordance with the local legislation and institutional requirements. The participants provided their written informed consent to participate in this study.

## Author contributions

YH: Conceptualization, Funding acquisition, Project administration, Writing – original draft, Writing – review & editing. MM: Methodology, Project administration, Supervision, Writing – review & editing. KO: Formal analysis, Writing – review & editing. TY: Investigation, Writing – review & editing. SA: Investigation, Writing – review & editing. HY: Formal analysis, Supervision, Writing – review & editing. HI: Resources, Supervision, Writing – review & editing. MI: Conceptualization, Supervision, Writing – review & editing.

## References

[1] Balestrini S Perozzi C Altamura C Vernieri F Luzzi S Bartolini M . Severe carotid stenosis and impaired cerebral hemodynamics can influence cognitive deterioration. Neurology. (2013) 80:2145–50. doi: 10.1212/WNL.0b013e318295d71a, PMID: 23624562

[2] Lal BK Dux MC Sikdar S Goldstein C Khan AA Yokemick J . Asymptomatic carotid stenosis is associated with cognitive impairment. J Vasc Surg. (2017) 66:1083–92. doi: 10.1016/J.JVS.2017.04.038 28712815

[3] Lazar RM Wadley VG Myers T Jones MR Heck DV Clark WM . Baseline cognitive impairment in patients with asymptomatic carotid stenosis in the CREST-2 trial. Stroke. (2021) 52:3855–63. doi: 10.1161/STROKEAHA.120.032972 34433306 PMC8608701

[4] Marshall RS Festa JR Cheung YK Chen R Pavol MA Derdeyn CP . Cerebral hemodynamics and cognitive impairment: baseline data from the RECON trial. Neurology. (2012) 78:250–5. doi: 10.1212/WNL.0b013e31824365d3, PMID: 22238418 PMC3280055

[5] Song P Fang Z Wang H Cai Y Rahimi K Zhu Y . Global and regional prevalence, burden, and risk factors for carotid atherosclerosis: a systematic review, meta-analysis, and modelling study. Lancet Glob Health. (2020) 8:e721–9. doi: 10.1016/S2214-109X(20)30117-0, PMID: 32353319

[6] De Weerd M Greving JP De Jong AWF Buskens E Bots ML. Prevalence of asymptomatic carotid artery stenosis according to age and sex systematic review and metaregression analysis. Stroke. (2009) 40:1105–13. doi: 10.1161/STROKEAHA.108.532218, PMID: 19246704

[7] O’Leary DH Polak JF Kronmal RA Kittner SJ Price TR Bond MG . Distribution and correlates of sonographically detected carotid artery disease in the cardiovascular health study. Stroke. (1992) 23:1752–60. doi: 10.1161/01.STR.23.12.1752 1448826

[8] Kirkpatrick AC Vincent AS Guthery L Prodan CI. Cognitive impairment is associated with medication nonadherence in asymptomatic carotid stenosis. Am J Med. (2014) 127:1243–6. doi: 10.1016/j.amjmed.2014.08.010, PMID: 25168078

[9] Veronica Witte A Kerti L Margulies DS Flöel A. Effects of resveratrol on memory performance, hippocampal functional connectivity, and glucose metabolism in healthy older adults. J Neurosci. (2014) 34:7862–70. doi: 10.1523/JNEUROSCI.0385-14.2014, PMID: 24899709 PMC6608268

[10] Griñán-Ferré C Bellver-Sanchis A Izquierdo V Corpas R Roig-Soriano J Chillón M . The pleiotropic neuroprotective effects of resveratrol in cognitive decline and Alzheimer’s disease pathology: from antioxidant to epigenetic therapy. Ageing Res Rev. (2021) 67:101271. doi: 10.1016/J.ARR.2021.101271, PMID: 33571701

[11] Wang N He J Pan C Wang J Ma M Shi X . Resveratrol activates autophagy via the AKT/mTOR Signaling pathway to improve cognitive dysfunction in rats with chronic cerebral Hypoperfusion. Front Neurosci. (2019) 13:1–14. doi: 10.3389/fnins.2019.00859, PMID: 31481868 PMC6710371

[12] Li H Wang J Wang P Rao Y Chen L. Resveratrol reverses the synaptic plasticity deficits in a chronic cerebral Hypoperfusion rat model. J Stroke Cerebrovasc Dis. (2016) 25:122–8. doi: 10.1016/j.jstrokecerebrovasdis.2015.09.004, PMID: 26456198

[13] Kang N Shi Y Song J Gao F Fan M Jin W . Resveratrol reduces inflammatory response and detrimental effects in chronic cerebral hypoperfusion by down-regulating stimulator of interferon genes/TANK-binding kinase 1/interferon regulatory factor 3 signaling. Front Aging Neurosci. (2022) 14:868484. doi: 10.3389/FNAGI.2022.868484, PMID: 35936778 PMC9354401

[14] Vingtdeux V Giliberto L Zhao H Chandakkar P Wu Q Simon JE . AMP-activated protein kinase signaling activation by resveratrol modulates amyloid-beta peptide metabolism. J Biol Chem. (2010) 285:9100–13. doi: 10.1074/JBC.M109.060061, PMID: 20080969 PMC2838330

[15] Park SJ Ahmad F Philp A Baar K Williams T Luo H . Resveratrol ameliorates aging-related metabolic phenotypes by inhibiting cAMP phosphodiesterases. Cell. (2012) 148:421–33. doi: 10.1016/j.cell.2012.01.017, PMID: 22304913 PMC3431801

[16] Hattori Y Okamoto Y Maki T Yamamoto Y Oishi N Yamahara K . Silent information regulator 2 homolog 1 counters cerebral hypoperfusion injury by deacetylating endothelial nitric oxide synthase. Stroke. (2014) 45:3403–11. doi: 10.1161/strokeaha.114.006265, PMID: 25213338

[17] Hattori Y Okamoto Y Nagatsuka K Takahashi R Kalaria RN Kinoshita M . SIRT1 attenuates severe ischemic damage by preserving cerebral blood flow. Neuroreport. (2015) 26:113–7. doi: 10.1097/wnr.0000000000000308 25634315

[18] Evans HM Howe PRC Wong RHX. Effects of resveratrol on cognitive performance, mood and cerebrovascular function in post-menopausal women; a 14-week randomised placebo-controlled intervention trial. Nutrients. (2017) 9:27. doi: 10.3390/nu9010027, PMID: 28054939 PMC5295071

[19] Thaung Zaw JJ Howe PR Wong RH. Long-term effects of resveratrol on cognition, cerebrovascular function and cardio-metabolic markers in postmenopausal women: a 24-month randomised, double-blind, placebo-controlled, crossover study. Clin Nutr. (2021) 40:820–9. doi: 10.1016/J.CLNU.2020.08.025, PMID: 32900519

[20] Zaw JJT Howe PRC Wong RHX. Sustained cerebrovascular and cognitive benefits of resveratrol in postmenopausal women. Nutrients. (2020) 12:828. doi: 10.3390/NU12030828, PMID: 32244933 PMC7146200

[21] Hattori Y Kakino Y Hattori Y Iwashita M Uchiyama H Noda K . Long-term resveratrol intake for cognitive and cerebral blood flow impairment in carotid artery stenosis/occlusion. J Stroke. (2024) 26:64–74. doi: 10.5853/JOS.2023.02733, PMID: 38326707 PMC10850448

[22] Jahromi AS Cinà CS Liu Y Clase CM. Sensitivity and specificity of color duplex ultrasound measurement in the estimation of internal carotid artery stenosis: a systematic review and meta-analysis. J Vasc Surg. (2005) 41:962–72. doi: 10.1016/j.jvs.2005.02.044, PMID: 15944595

[23] Giannopoulos S Katsanos AH Tsivgoulis G Marshall RS. Statins and cerebral hemodynamics. J Cereb Blood Flow Metab. (2012) 32:1973–6. doi: 10.1038/JCBFM.2012.122, PMID: 22929438 PMC3494001

[24] Pauls MMH Moynihan B Barrick TR Kruuse C Madigan JB Hainsworth AH . The effect of phosphodiesterase-5 inhibitors on cerebral blood flow in humans: a systematic review. J Cereb Blood Flow Metab. (2018) 38:189–203. doi: 10.1177/0271678X17747177, PMID: 29256324 PMC5951021

[25] Etminan M Gill S Samii A. Effect of non-steroidal anti-inflammatory drugs on risk of Alzheimer’s disease: systematic review and meta-analysis of observational studies. BMJ. (2003) 327:128–31. doi: 10.1136/BMJ.327.7407.128 12869452 PMC165707

[26] Iguchi S Moriguchi T Yamazaki M Hori Y Koshino K Toyoda K . System evaluation of automated production and inhalation of 15 O-labeled gaseous radiopharmaceuticals for the rapid 15 O-oxygen PET examinations. EJNMMI Phys. (2018) 5:37. doi: 10.1186/s40658-018-0236-5, PMID: 30569426 PMC6300454

[27] Kudomi N Hayashi T Teramoto N Watabe H Kawachi N Ohta Y . Rapid quantitative measurement of CMRO2 and CBF by dual administration of ^15^O-labeled oxygen and water during a single PET scan -a validation study and error analysis in anesthetized monkeys. J Cereb Blood Flow Metab. (2005) 25:1209–24. doi: 10.1038/sj.jcbfm.9600118, PMID: 15874976

[28] Kudomi N Choi E Yamamoto S Watabe H Kim KM Shidahara M . Development of a GSO detector assembly for a continuous blood sampling system. IEEE Trans Nucl Sci. (2003) 50:70–3. doi: 10.1109/TNS.2002.807869

[29] Iida H Jones T Miura S. Modeling approach to eliminate the need to separate arterial plasma in oxygen-15 inhalation positron emission tomography. J Nucl Med. (1993) 34:1333–40. PMID: 8326395

[30] Thal LJ Ferguson JM Mintzer J Raskin A Targum SD. A 24-week randomized trial of controlled-release physostigmine in patients with Alzheimer’s disease. Neurology. (1999) 52:1146–52. doi: 10.1212/WNL.52.6.1146, PMID: 10214735

[31] Wilkinson D Doody R Helme R Taubman K Mintzer J Kertesz A . Donepezil in vascular dementia: a randomized, placebo-controlled study. Neurology. (2003) 61:479–86. doi: 10.1212/01.WNL.0000078943.50032.FC 12939421

[32] Winblad B Cummings J Andreasen N Grossberg G Onofrj M Sadowsky C . A six-month double-blind, randomized, placebo-controlled study of a transdermal patch in Alzheimer’s disease--rivastigmine patch versus capsule. Int J Geriatr Psychiatry. (2007) 22:456–67. doi: 10.1002/GPS.1788 17380489

[33] Bo M Massaia M Speme S Cappa G Strumia K Cerrato P . Risk of cognitive decline in older patients after carotid endarterectomy: an observational study. J Am Geriatr Soc. (2006) 54:932–6. doi: 10.1111/J.1532-5415.2006.00787.X 16776788

[34] Ogasawara K Yamadate K Kobayashi M Endo H Fukuda T Yoshida K . Postoperative cerebral hyperperfusion associated with impaired cognitive function in patients undergoing carotid endarterectomy. J Neurosurg. (2005) 102:38–44. doi: 10.3171/JNS.2005.102.1.0038, PMID: 15658094

[35] Heyer EJ Sharma R Rampersad A Winfree CJ Mack WJ Solomon RA . A controlled prospective study of neuropsychological dysfunction following carotid endarterectomy. Arch Neurol. (2002) 59:217–22. doi: 10.1001/ARCHNEUR.59.2.217, PMID: 11843692 PMC2435245

[36] Heyer EJ Adams DC Solomon RA Todd GJ Quest DO McMahon DJ . Neuropsychometric changes in patients after carotid endarterectomy. Stroke. (1998) 29:1110–5. doi: 10.1161/01.STR.29.6.1110, PMID: 9626280 PMC2435204

[37] Gaunt ME Martin PJ Smith JL Rimmer T Cherryman G Ratliff DA . Clinical relevance of intraoperative embolization detected by transcranial Doppler ultrasonography during carotid endarterectomy: a prospective study of 100 patients. Br J Surg. (1994) 81:1435–9. doi: 10.1002/bjs.1800811009, PMID: 7820463

[38] Ogasawara K Inoue T Kobayashi M Fukuda T Komoribayashi N Saitoh H . Cognitive impairment associated with intraoperative and postoperative hypoperfusion without neurologic deficits in a patient undergoing carotid endarterectomy. Surg Neurol. (2006) 65:577–80. doi: 10.1016/J.SURNEU.2005.07.011, PMID: 16720178

[39] De Rango P Caso V Leys D Paciaroni M Lenti M Cao P. The role of carotid artery stenting and carotid endarterectomy in cognitive performance: a systematic review. Stroke. (2008) 39:3116–27. doi: 10.1161/STROKEAHA.108.518357, PMID: 18723423

[40] Gorelick PB Scuteri A Black SE Decarli C Greenberg SM Iadecola C . Vascular contributions to cognitive impairment and dementia: a statement for healthcare professionals from the American Heart Association/American Stroke Association. Stroke. (2011) 42:2672–713. doi: 10.1161/STR.0b013e3182299496, PMID: 21778438 PMC3778669

[41] Toth P Tarantini S Tucsek Z Ashpole NM Sosnowska D Gautam T . Resveratrol treatment rescues neurovascular coupling in aged mice: role of improved cerebromicrovascular endothelial function and downregulation of NADPH oxidase. Am J Physiol Heart Circ Physiol. (2014) 306:H299–308. doi: 10.1152/ajpheart.00744.2013, PMID: 24322615 PMC3920140

[42] Wong RHX Howe PRC Buckley JD Coates AM Kunz I Berry NM. Acute resveratrol supplementation improves flow-mediated dilatation in overweight/obese individuals with mildly elevated blood pressure. Nutr Metab Cardiovasc Dis. (2011) 21:851–6. doi: 10.1016/j.numecd.2010.03.003, PMID: 20674311

[43] Wilson T Knight TJ Beitz DC Lewis DS Engen RL. Resveratrol promotes atherosclerosis in hypercholesterolemic rabbits. Life Sci. (1996) 59:PL15–21. doi: 10.1016/0024-3205(96)00260-3, PMID: 8684261

[44] Fan D Liu C Zhang Z Huang K Wang T Chen S . Progress in the preclinical and clinical study of resveratrol for vascular metabolic disease. Molecules. (2022) 27:27. doi: 10.3390/MOLECULES27217524, PMID: 36364370 PMC9658204

[45] Baur JA Sinclair DA. Therapeutic potential of resveratrol: the in vivo evidence. Nat Rev Drug Discov. (2006) 5:493–506. doi: 10.1038/NRD2060 16732220

[46] Calamini B Ratia K Malkowski MG Cuendet M Pezzuto JM Santarsiero BD . Pleiotropic mechanisms facilitated by resveratrol and its metabolites. Biochem J. (2010) 429:273–82. doi: 10.1042/BJ20091857, PMID: 20450491 PMC3265359

[47] Man AWC Li H Xia N. The role of Sirtuin 1 in regulating endothelial function, arterial Remodeling and vascular aging. Front Physiol. (2019) 10:1173. doi: 10.3389/FPHYS.2019.01173, PMID: 31572218 PMC6751260

[48] Mattagajasingh I Kim CS Naqvi A Yamamori T Hoffman TA Jung SB . SIRT1 promotes endothelium-dependent vascular relaxation by activating endothelial nitric oxide synthase. Proc Natl Acad Sci USA. (2007) 104:14855–60. doi: 10.1073/pnas.0704329104, PMID: 17785417 PMC1976244

[49] Choi S Saxena N Dhammu T Khan M Singh AK Singh I . Regulation of endothelial barrier integrity by redox-dependent nitric oxide signaling: implication in traumatic and inflammatory brain injuries. Nitric Oxide. (2019) 83:51–64. doi: 10.1016/J.NIOX.2018.12.007, PMID: 30590116 PMC6546177

[50] Walker J Schueller K Schaefer LM Pignitter M Esefelder L Somoza V. Resveratrol and its metabolites inhibit pro-inflammatory effects of lipopolysaccharides in U-937 macrophages in plasma-representative concentrations. Food Funct. (2014) 5:74–84. doi: 10.1039/C3FO60236B, PMID: 24257684

[51] Turner RS Thomas RG Craft S Van Dyck CH Mintzer J Reynolds BA . A randomized, double-blind, placebo-controlled trial of resveratrol for Alzheimer disease. Neurology. (2015) 85:1383–91. doi: 10.1212/WNL.0000000000002035, PMID: 26362286 PMC4626244

[52] Popat R Plesner T Davies F Cook G Cook M Elliott P . A phase 2 study of SRT501 (resveratrol) with bortezomib for patients with relapsed and or refractory multiple myeloma. Br J Haematol. (2013) 160:714–7. doi: 10.1111/bjh.12154 23205612

[53] Louis Bresson J Burlingame B Dean T Fairweather-Tait S Heinonen M Ildico Hirsch-Ernst K . Safety of synthetic trans-resveratrol as a novel food pursuant to regulation (EC) no 258/97. EFSA J. (2016) 14:4368. doi: 10.2903/J.EFSA.2016.4368

